# Stress Fracture in Athletes: A Practical Approach

**DOI:** 10.3390/jcm15083077

**Published:** 2026-04-17

**Authors:** Federica Presutti, Stefano Paoletti, Francesca Conte, Andrea Demeco, Felice Sirico, Rossana Gnasso, Marco Vecchiato, Veronica Baioccato, Alessandro Corsini, Simone Cerciello, Matteo Guzzini, Stefano Palermi

**Affiliations:** 1 Departmental Faculty of Medicine and Surgery, UniCamillus Saint Camillus International University of Health Sciences, 00131 Rome, Italy; u.020636@students.unicamillus.org (F.P.); stemeds26@gmail.com (S.P.); simone.cerciello@unicamillus.org (S.C.); matteo.guzzini@unicamillus.org (M.G.); 2 Cardiology and Sport Medicine Department, Ospedale Riabilitativo di Alta Specializzazione di Motta di Livenza, 31045 Treviso, Italy; francesca.conte.medsport@gmail.com; 3 Department of Medical and Surgical Sciences, University of Catanzaro “Magna Graecia”, 88100 Catanzaro, Italy; andreademeco@hotmail.it; 4 Public Health Department, University of Naples Federico II, 80123 Naples, Italy; sirico.felice@unina.it (F.S.); gnasso.rossana@unina.it (R.G.); 5 Sports and Exercise Medicine Division, University of Padova, 35122 Padova, Italy; marcovecchiato.md@gmail.com (M.V.); veronica.baioccato@aopd.veneto.it (V.B.); 6 Department of Theoretical and Applied Sciences, eCampus University, 22060 Novedrate, Italy; 7 Genoa Cricket and Football Club, 16155 Genova, Italy; dottor@alessandrocorsini.it

**Keywords:** stress fractures, return to play, rehabilitation, sports

## Abstract

Stress fractures (SFs) are a common overuse injury in athletes and represent the severe end of the bone stress injury (BSI) continuum. They result from repetitive mechanical loading exceeding the bone’s capacity for adaptation and are associated with impaired performance, prolonged time away from sport, and risk of recurrence if not appropriately managed. This narrative review provides a clinically oriented synthesis of current evidence on the epidemiology, pathophysiology, risk factors, diagnosis, management, and prevention of SFs in athletes. Particular emphasis is placed on modifiable contributors, including training load errors, neuromuscular fatigue, and low energy availability within the framework of Relative Energy Deficiency in Sport (RED-S). Diagnostic evaluation is discussed using a stepwise clinical approach integrating history, physical examination, targeted laboratory assessment, and imaging, with magnetic resonance imaging (MRI) as the reference standard for early detection and severity grading. Management is presented through a risk-based framework combining MRI severity and anatomical site classification to guide treatment decisions and return-to-sport pathways. While most low-risk SFs respond to conservative strategies, high-risk lesions require closer monitoring and, in selected cases, early surgical consideration. This review proposes a practical clinical framework to support decision-making in athletes with suspected or confirmed SFs, aiming to improve early diagnosis, optimize management, and reduce recurrence risk in sports medicine practice.

## 1. Introduction

Stress fractures (SFs) are part of a continuum of bone stress injuries (BSIs) caused by repetitive mechanical loading that exceeds the bone’s capacity for physiological adaptation and remodeling. This spectrum ranges from early, potentially reversible stress reactions to established cortical fractures, and is particularly prevalent in athletes exposed to high training volumes or repetitive impact loading [1].

SFs typically occur in otherwise healthy individuals subjected to sustained biomechanical stress, most commonly athletes and military personnel. They account for approximately 5–10% of all sports-related injuries, with reported prevalence rates around 0.7–1.0% in recreational athletic populations [2]. The development of SFs is multifactorial and reflects the interaction between extrinsic factors—such as rapid increases in training load, surface characteristics, and footwear—and intrinsic factors including biomechanical alignment, bone mineral density, hormonal status, and nutritional adequacy [3].

Despite increasing awareness and a growing body of literature on BSIs, the clinical management of SFs remains challenging. In particular, there is considerable heterogeneity in diagnostic pathways, risk stratification, and return-to-sport decisions, often reflecting variability in clinical practice rather than standardized approaches [4]. While magnetic resonance imaging (MRI) has improved early detection and severity assessment, and anatomical risk classification has been proposed to guide prognosis, these elements are often applied in isolation. This lack of integration may contribute to delayed diagnosis, suboptimal management, and an increased risk of recurrence, particularly among high-performance athletes [4].

Therefore, the aim of this narrative review is to provide a clinically oriented and integrative framework for the evaluation and management of SFs in athletes. Specifically, this review combines current evidence on MRI-based severity grading and anatomical risk classification into a practical decision-making approach, with a focus on diagnosis, treatment strategies, and safe return to sport. Particular attention is given to modifiable contributors such as training errors, biomechanical factors, and low energy availability within the framework of Relative Energy Deficiency in Sport (RED-S). SFs and BSIsSFs in athletes.

## 2. Methods

This work was designed as a narrative, clinically oriented review aimed at providing a practical synthesis of current evidence on SFs and BSIs in athletes. A formal systematic review methodology was not applied, as the primary objective was to integrate available evidence into a clinically applicable framework rather than to perform a quantitative synthesis. A literature search was conducted using the electronic databases PubMed and Scopus to identify relevant studies published up to January 2026. The search strategy included combinations of the following keywords: “stress fractures”, “bone stress injuries”, “athletes”, “sports injuries”, “MRI”, “rehabilitation”, and “relative energy deficiency in sport (RED-S)”. Studies were selected based on their relevance to clinical practice and their contribution to key domains, including epidemiology, pathophysiology, diagnosis, imaging, treatment, rehabilitation, and prevention. Priority was given to recent systematic reviews and meta-analyses, international consensus statements, and high-quality prospective or retrospective clinical studies. Earlier landmark studies were also included when considered essential for contextual understanding. Articles were excluded if they focused exclusively on non-athletic populations, consisted of isolated case reports without broader clinical implications, or lacked sufficient methodological clarity. Given the heterogeneity of the available literature, no formal risk-of-bias assessment or statistical synthesis was performed. Instead, the selected evidence was critically appraised and integrated to develop a practical, decision-oriented framework for clinicians managing athletes with suspected or confirmed SFs.

## 3. Epidemiology

The epidemiology of SFs in athletes reflects a multifactorial interaction between intrinsic and extrinsic determinants, including sport-specific loading patterns, sex, age, training volume, and individual biomechanical characteristics. Consistently higher incidence rates have been reported in women, endurance athletes, and military personnel, largely due to cumulative mechanical loading and the frequent coexistence of predisposing factors such as low energy availability and RED-S [3].

SFs may involve virtually any skeletal site; however, their anatomical distribution varies substantially according to sport-specific biomechanical demands (Table 1). Endurance and impact-based activities, including long-distance running and dance, predominantly affect the tibia and metatarsals, whereas sports characterized by rapid acceleration, cutting, and directional changes—such as football, basketball, and tennis—are associated with a broader injury spectrum involving the navicular bone, pars interarticularis, and fifth metatarsal [5].

Lower-limb bones account for the majority of SFs, representing over 40% of injuries in large military cohorts [6]. Among runners, the tibia is the most frequently involved site (approximately one-third of cases), followed by the tarsal bones, metatarsals, and femur [7]. Sport-specific patterns have also been described in gymnasts, who commonly develop pars interarticularis stress injuries related to repetitive lumbar hyperextension, and in rowers and golfers, in whom rib stress fractures predominate [8].

Sex-related differences further influence epidemiological patterns. Female athletes demonstrate higher rates of pelvic and metatarsal SFs, likely related to lower bone mineral density, hormonal perturbations, and a higher prevalence of low energy availability, whereas men exhibit comparatively lower rates of SFs in sites such as the fibula [9]. Age and skeletal maturity also modify risk, with adolescents being particularly vulnerable due to ongoing bone development and open physes. High-risk sports among adolescents include basketball, gymnastics, and cheerleading, especially among females [10].

Military populations consistently report some of the highest SF incidence rates, largely attributable to abrupt increases in physical load during basic training. Large prospective studies have documented SF occurrence in up to 15–20% of female recruits and 5–6% of male recruits, with markedly higher incidence rates per person-year compared with athletic cohorts [11].

Risk factors for SFs are traditionally categorized as extrinsic or intrinsic, both of which influence the bone’s ability to tolerate repetitive mechanical loading and to adapt effectively to sport-specific demands (Table 2) [12].

Extrinsic risk factors primarily relate to training characteristics and environmental conditions. Sudden increases in training volume, intensity, or frequency—particularly in the absence of adequate progression or recovery—represent one of the strongest and most consistently reported predictors of SFs, as they exceed the bone’s remodeling capacity and promote microdamage accumulation [4]. Training on hard, irregular, or sloped surfaces further amplifies mechanical stress, while inappropriate or excessively worn footwear alters load distribution and impact attenuation [13]. Evidence from military and athletic populations suggests that optimized footwear and shock-absorbing insoles may reduce the incidence of lower-limb stress injuries, particularly in high-load environments, although their preventive effect is not universal [14]. Inadequate recovery between training sessions is another contributor, as insufficient rest impairs bone repair and facilitates progression from a stress reaction to an overt fracture [4]. A history of previous SFs is among the strongest predictors of recurrence, often reflecting unresolved biomechanical issues, persistent low energy availability, or chronic training errors [15].

Intrinsic risk factors encompass biological, anatomical, and metabolic characteristics. Female sex has been consistently associated with increased SF risk, mediated by lower average bone mineral density, menstrual dysfunction, and a higher prevalence of low energy availability [16]. The former concept of the Female Athlete Triad has been expanded into the broader RED-S framework, which includes multisystem consequences of low energy availability and is now recognized as one of the most important intrinsic determinants of bone stress injuries [9].

Biomechanical and anatomical factors further modulate injury risk. Pes cavus or pes planus, excessive subtalar pronation, altered hip rotation, and reduced tibial cross-sectional geometry have all been associated with increased SF susceptibility, particularly in runners and military recruits [17]. From a nutritional perspective, insufficient intake of calcium, vitamin D, and protein negatively affects bone turnover and mineralization. Although supplementation is not universally protective, combined calcium and vitamin D supplementation has been shown to reduce SF incidence in at-risk populations, especially in individuals with documented deficiencies or restrictive dietary patterns [18]. However, epidemiological findings remain heterogeneous across studies, largely due to variations in sport-specific exposure, injury definitions, and surveillance methodologies.

## 4. Pathophysiology

The pathophysiology of SFs, increasingly recognized as part of the broader spectrum of BSIs, results from a complex interaction among repetitive mechanical loading, bone remodeling dynamics, neuromuscular function, hormonal regulation, and nutritional status. When the cumulative mechanical load exceeds the bone’s adaptive capacity, structural integrity is progressively compromised, predisposing athletes to injury and delayed healing [19].

### 4.1. Bone Remodeling Imbalance and Microdamage Accumulation

Under physiological conditions, bone adapts to mechanical stimuli through a tightly regulated remodeling process characterized by osteoclastic resorption followed by osteoblastic bone formation, as described by Wolff’s law [20]. However, excessive loading frequency, magnitude, or strain rate may overwhelm this adaptive response. In such circumstances, microdamage accumulates faster than it can be repaired, resulting in a transient reduction in bone stiffness and strength due to a predominance of resorptive activity—a phenomenon often referred to as remodeling imbalance or remodeling lag [20]. Persistent loading under these conditions allows microdamage to propagate through cortical and trabecular structures, ultimately leading to overt stress fracture formation [21].

### 4.2. From Stress Reaction to Stress Fracture

BSIs exist along a biological continuum. Early-stage lesions, commonly termed stress reactions, are characterized by bone marrow edema and microscopic trabecular disruption without a visible fracture line on imaging. Continued mechanical exposure promotes cortical involvement and the development of a discrete fracture line, marking progression to a true stress fracture [22].

### 4.3. Mechanical Loading Characteristics and Neuromuscular Fatigue

The mechanical environment plays a critical role in SF development. Strain rate, rather than load magnitude alone, is a key determinant of microdamage formation, particularly during high-impact or plyometric activities [23]. SFs are commonly categorized according to the predominant mechanical force involved: compressive (e.g., posteromedial tibial SFs), tensile (e.g., anterior tibial cortex SFs), or shear-related injuries. Tensile-side lesions are especially prone to delayed healing due to the bone’s limited tolerance to tensile stress [24].

Skeletal muscle function provides an important protective mechanism by attenuating impact forces and reducing load transmission to bone. Neuromuscular fatigue diminishes this protective capacity, leading to increased bone strain, particularly in the tibia, femur, and metatarsals. This mechanism helps explain the association between SFs and late-session training, insufficient recovery, and consecutive days of high-intensity exercise [25].

### 4.4. Hormonal and Metabolic Influences

Hormonal status is a central determinant of bone turnover. Estrogens suppress osteoclastic activity and promote bone mineral density; consequently, hypoestrogenism—commonly observed in athletes with low energy availability—increases bone resorption and SF susceptibility [26]. Within this framework, RED-S represents a key contributor to impaired bone metabolism and increased injury risk” [9].

### 4.5. Nutritional Factors and Emerging Hypotheses

Adequate intake of calcium, vitamin D, and protein is essential for bone mineralization and collagen synthesis. Deficiencies in these nutrients negatively affect bone quality and increase vulnerability to BSIs, although supplementation appears most beneficial in athletes with documented deficiencies rather than as a universal preventive strategy [18].

An additional, less established mechanism is the bone ischemia hypothesis, which suggests that repetitive mechanical loading may impair local microvascular perfusion, promoting osteoclastic activity and structural weakening. While biologically plausible, evidence supporting this mechanism remains limited and its clinical relevance is yet to be fully clarified [27].

## 5. Diagnosis

### 5.1. History and Physical Examination

The diagnostic work-up of a suspected SF starts with a detailed clinical history and a focused physical examination. Athletes typically report an insidious onset of localized pain that is initially activity-related and progressively worsens with continued loading, eventually persisting during daily activities or at rest in more advanced stages [28].

A structured history should systematically explore training characteristics and recent load variations, including abrupt changes in volume, intensity, frequency, training surface, or footwear. Particular attention should be paid to recent training spikes occurring within the preceding 2–6 weeks, which are consistently associated with SF onset [29]. Additional key elements include prior SFs or overuse injuries, footwear type and wear pattern, and symptoms suggestive of low energy availability or RED-S, such as fatigue, weight loss, recurrent injuries, or menstrual disturbances in female athletes [9,26]. Nutritional habits, including restrictive diets or inadequate intake of calcium and vitamin D, should also be assessed [30].

Physical examination aims to identify focal bone tenderness and contributory biomechanical factors. Point tenderness directly over the involved bone is a hallmark finding and typically differentiates SFs from soft-tissue injuries, which often improve with warm-up. Local swelling or warmth may be present but is frequently minimal, particularly in early-stage injuries [31]. A global biomechanical assessment should include comparison with the contralateral limb and evaluation of the kinetic [9,26] chain, with attention to lower-limb alignment, hip and pelvic control, and foot morphology (e.g., pes cavus, pes planus, excessive pronation or supination) [3].

Several clinical tests may support the suspicion of SFs, including the single-leg hop test for tibial or femoral lesions, the fulcrum test for femoral shaft injuries, and lumbar extension tests for pars interarticularis involvement, particularly in gymnasts [32,33]. High-risk or deep SFs, such as those involving the femoral neck, may be difficult to localize on examination and can present with nonspecific findings, including antalgic gait or pain elicited by hip rotation or straight-leg raise [34]. Beyond supporting diagnosis, a structured clinical assessment is essential for identifying modifiable risk factors and guiding individualized load management strategies.

### 5.2. Laboratory Evaluation

Laboratory testing is not routinely required for athletes presenting with a first, uncomplicated SF but becomes appropriate in selected clinical scenarios, including recurrent or multiple SFs, atypical or high-risk locations (e.g., femoral neck, pelvis, ribs), delayed healing, suspected RED-S, or adherence to restrictive dietary patterns [9,35].

Based on current bone health recommendations and the International Olympic Committee (IOC) consensus on RED-S, a targeted laboratory panel should include assessment of vitamin D status (25-OH vitamin D), serum calcium and phosphate, parathyroid hormone, and markers of bone turnover such as alkaline phosphatase [9,21]. When endocrine dysfunction is suspected, additional evaluation may include thyroid function tests, gonadotropins, estradiol in female athletes, total and free testosterone in males, prolactin, and morning cortisol [36]. Nutritional and metabolic screening may be expanded to include complete blood count, ferritin and iron studies, albumin, vitamin B12, and folate, particularly in endurance athletes or those with low energy availability [37].

Identification of metabolic or endocrine abnormalities should prompt further investigations, such as dual-energy X-ray absorptiometry (DXA), and early involvement of a multidisciplinary team including sports nutritionists and endocrinologists. Additional biomarkers of bone turnover, such as bone-specific alkaline phosphatase and osteocalcin, may provide further insight into bone remodeling dynamics, although their routine clinical use remains limited.

### 5.3. Imaging

Imaging is central to confirming the diagnosis of SFs, grading injury severity, and guiding treatment decisions and return-to-sport timelines. Clinical assessment alone may underestimate injury severity, particularly in early-stage BSIs, underscoring the importance of timely imaging [31,38].

#### 5.3.1. Plain Radiography

Plain radiographs are commonly used as a first-line investigation due to availability and low cost, but they demonstrate very low sensitivity in early BSIs, with radiographic signs such as periosteal reaction, cortical lucency, or callus formation typically appearing only after 2–6 weeks of symptoms [7,31]. A normal radiograph does not exclude an SF but may help rule out acute fractures, tumors, infection, or gross structural abnormalities.

#### 5.3.2. Bone Scintigraphy

Technetium-99 m bone scintigraphy has high sensitivity for detecting increased bone turnover and may identify lesions earlier than radiography. However, its limited specificity, radiation exposure, and inferior anatomical resolution compared with magnetic resonance imaging (MRI) have restricted its use to selected or problem-solving cases [39].

#### 5.3.3. Computed Tomography

Computed tomography (CT) provides excellent visualization of cortical bone and is particularly useful for defining fracture lines, evaluating pars interarticularis injuries, and differentiating SFs from alternative diagnoses such as osteoid osteoma or osteomyelitis. Its inability to detect bone marrow edema and associated radiation exposure limits its role in early-stage BSIs [38].

#### 5.3.4. Magnetic Resonance Imaging

MRI is the current gold standard for SF diagnosis due to its high sensitivity and specificity, its ability to detect early bone marrow edema and assess adjacent soft tissues, and its lack of ionizing radiation [40,41]. Fluid-sensitive sequences (T2-weighted or STIR) highlight edema, whereas T1-weighted images allow identification of hypointense fracture lines and cortical disruption. MRI is particularly indicated in high-risk anatomical sites such as the femoral neck, navicular bone, and anterior tibial cortex, even if CT can play an interesting role in such particular locations. Importantly, imaging findings should always be interpreted in conjunction with clinical symptoms, as marrow edema may persist despite clinical recovery [42].

MRI-based grading systems, such as the Kaeding–Miller classification, are commonly used to stratify injury severity and guide management, although no single system has universal acceptance [43] (Table 3).

### 5.4. Bone Mineral Density Assessment

DXA assessment is not routinely indicated for all athletes with SFs but should be considered in cases of recurrent injuries, high-risk fracture sites, suspected RED-S, menstrual dysfunction, abnormal laboratory findings, or significant weight loss [9,36]. DXA provides information on bone mineral density, body composition, and Z-scores relevant to athletic populations. Low Z-scores should prompt targeted nutritional and endocrine interventions and reinforce the need for a multidisciplinary management strategy.

### 5.5. Differential Diagnosis

The differential diagnosis of stress fractures includes several conditions presenting with activity-related pain, such as periostitis, tendinopathies, chronic exertional compartment syndrome, and muscle strains. In some cases, more serious conditions, such as bone tumors or infections, should be considered, particularly in the presence of atypical symptoms, nocturnal pain, or systemic signs. Accurate differentiation is essential to avoid delayed diagnosis and inappropriate management.

## 6. Therapy

Management of SFs encompasses conservative and surgical strategies, selected according to injury severity, anatomical risk, athlete characteristics, and performance demands. The primary goals of treatment are to achieve reliable bone healing, minimize the risk of recurrence, and enable a safe, progressive return to sport [44,45].

### 6.1. Non-Surgical Management

Conservative treatment represents the first-line approach for most SFs, particularly those involving low-risk anatomical sites. Core principles include appropriate load modification, symptom-guided activity restriction, correction of contributing risk factors, and progressive reconditioning [46].

#### 6.1.1. Load Modification and Relative Rest

Initial management typically involves a period of relative rest, avoiding activities that reproduce pain. Although timelines vary according to injury severity and location, a symptom-guided rest period of approximately 6–8 weeks is commonly required. Return to impact activity should be considered only after the athlete has been pain-free for at least 10–14 days during daily activities and low-impact exercise [5].

During the unloading phase, cardiovascular fitness may be maintained using low-impact modalities such as cycling, swimming, deep-water running, or anti-gravity treadmill training, when available [47]. Temporary immobilization with walking boots, braces, or external supports may be appropriate in selected lower-limb SFs, particularly when pain persists during normal ambulation [48].

#### 6.1.2. Nutritional and Pharmacological Considerations

Adequate nutritional support is a cornerstone of SF management. Calcium and vitamin D supplementation should not be prescribed routinely but instead considered in athletes with inadequate dietary intake, documented deficiency, restrictive diets, or suspected low energy availability within the RED-S framework [49]. While supplementation may reduce SF incidence in deficient populations, evidence supporting a direct effect on fracture healing remains limited [50].

The use of bisphosphonates in athletes with SFs remains controversial. Although short-term pain reduction has been reported in small studies, these agents suppress bone remodeling and may theoretically impair microdamage repair, in addition to carrying age- and fertility-related safety concerns. Consequently, bisphosphonates are not recommended for routine management or primary prevention of SFs and should be considered only in exceptional cases under specialist supervision [51,52].

Recombinant parathyroid hormone (teriparatide) has been investigated in selected cases of delayed union or high-risk fractures, but current evidence is insufficient to support routine use in athletic populations [4].

Extracorporeal shock wave therapy (ESWT) has shown potential benefits in refractory SFs and delayed unions, particularly involving the tibia or metatarsals. However, available data are limited and heterogeneous, and ESWT should currently be regarded as an adjunctive option rather than standard care [27,53].

#### 6.1.3. Biomechanical Optimization and Recovery

Correction of biomechanical contributors is essential to reduce the risk of recurrence. This may include footwear optimization, shock-absorbing insoles, custom orthotics, and targeted strengthening of kinetic-chain deficits [12,37]. Adequate sleep, psychological stress management, and structured recovery are increasingly recognized as important modulators of bone remodeling and injury recovery [21].

Optimal conservative management is best achieved through a multidisciplinary approach involving sports physicians, physiotherapists, strength and conditioning specialists, sports dietitians, and, when indicated, sports psychologists and endocrinologists [9].

### 6.2. Surgical Management

Surgical management should be reserved for selected SFs characterized by a high risk of progression, displacement, or non-union, or for lesions that fail to respond to appropriately conducted conservative treatment. According to recent international consensus, indications for surgery are primarily determined by anatomical site and biomechanical loading characteristics rather than imaging severity alone, with particular concern for femoral neck (tension side), navicular bone, anterior tibial cortex, and proximal fifth metatarsal stress fractures. In these locations, early surgical stabilization may reduce the risk of catastrophic complications and facilitate a more predictable return to sport. Conversely, routine surgical intervention is not recommended for low-risk sites or early-stage bone stress injuries, where conservative management remains highly effective. Surgical decision-making should therefore be individualized, integrated within a multidisciplinary framework, and aligned with athlete-specific performance demands and long-term skeletal health considerations [5].

### 6.3. Rehabilitation and Return-to-Sport Pathways

Rehabilitation following SFs should be structured, individualized, and based primarily on two determinants: (1) injury severity as assessed by MRI grading and (2) anatomical risk classification (low-risk versus high-risk sites). Recent international consensus statements support a simplified dichotomous risk stratification, as intermediate-risk categories have limited clinical utility [54] (Table 4).

Low-risk SFs typically respond well to conservative rehabilitation. Initial management focuses on pain control and load reduction, with progression to impact activities only after complete symptom resolution. A gradual return-to-sport protocol should begin with reduced intensity and volume, followed by incremental increases over several weeks, guided by symptom response and functional testing [55]. High-risk SFs require a more cautious approach, often involving periods of absolute rest or protected weight-bearing, frequent clinical and imaging follow-up, and early orthopedic consultation. Surgical management should be considered in cases of persistent symptoms, radiographic progression, displaced fractures, or recurrent injuries at the same site [27,56].

**Figure 1:**
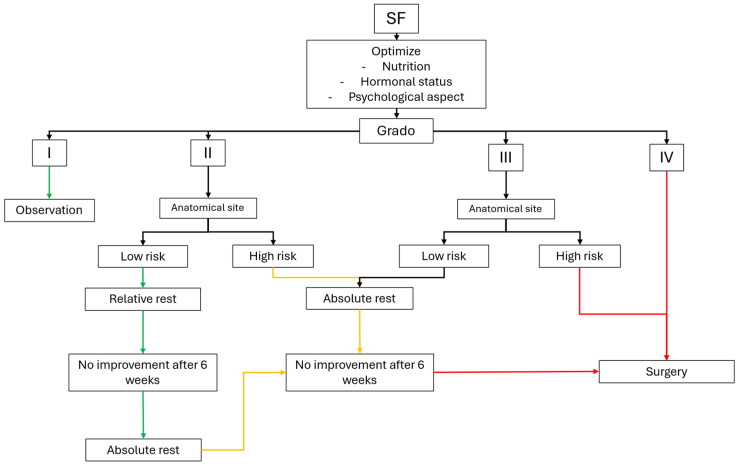
**Clinical decision-making algorithm for the management of suspected stress fractures (SFs) in athletes.** The proposed pathway integrates MRI severity grading with anatomical risk classification to guide initial management. Low-grade injuries (MRI grades I–II) are managed conservatively, with escalation of rest and further imaging if symptoms persist. Higher-grade lesions (MRI grades III–V), particularly at high-risk anatomical sites, require stricter load restriction, closer monitoring, and early surgical consideration. Optimization of modifiable risk factors—including training load, nutrition, equipment, and hormonal status—should be addressed at all stages of management.

Across all SF types, return-to-sport decisions must remain individualized, symptom-guided, and integrated within a broader load-management strategy to minimize recurrence risk (Figure 1).

## 7. Prevention

Preventing SFs is a central objective for athletes exposed to repetitive or high-impact loading. Although numerous preventive strategies have been proposed, only a limited number are supported by consistent evidence. Effective prevention requires a multifactorial approach targeting modifiable intrinsic and extrinsic risk factors, with particular emphasis on training load management, biomechanical optimization, nutritional adequacy, and early identification of RED-S [3,4].

### 7.1. Training Load Management

Avoidance of rapid increases in training load is the most consistently supported preventive strategy for SFs. Sudden spikes in volume, intensity, or frequency are strongly associated with bone stress injuries, particularly in runners, dancers, and military populations [57].

Key principles of load management include gradual progression of training stimuli, incorporation of structured recovery days, and avoidance of prolonged sequences of consecutive high-impact sessions. Although the commonly cited “10–15% rule” lacks robust experimental validation, progressive and individualized load increases remain a pragmatic approach in clinical practice [37,58]. Early recognition of warning symptoms—such as focal bone pain, nocturnal pain, or worsening pain with continued activity—is essential to prevent progression along the bone stress injury continuum.

### 7.2. Footwear and Biomechanics

Appropriate footwear selection helps mitigate repetitive bone loading. Key features include adequate midsole cushioning, appropriate stability for overpronation, sufficient forefoot flexibility, and regular replacement in runners, typically after 500–800 km [7,37].

Cushioned insoles and orthotics have demonstrated preventive benefits in military populations, particularly for tibial and metatarsal stress fractures, although results are less consistent in athletic cohorts [59,60]. Biomechanical assessment should therefore be individualized and integrated with strength and movement-quality interventions rather than relying solely on passive supports.

### 7.3. Nutritional and Hormonal Optimization

Adequate nutrition is fundamental for bone health and adaptive remodeling. Low energy availability represents one of the strongest intrinsic predictors of SFs across athletic populations [61].

Calcium and vitamin D supplementation should not be prescribed universally but should be considered for athletes with inadequate dietary intake, documented deficiency, indoor training, or seasonal risk factors. A randomized controlled trial in military recruits demonstrated a reduction in SF incidence with daily supplementation of calcium (2000 mg) and vitamin D (800 IU), particularly in individuals with low baseline levels [62]. Adequate intake of protein, iron, vitamin K, and overall energy availability further supports bone turnover and adaptation to training loads [63].

### 7.4. Strength Training and Conditioning

Isolated stretching programs have not been shown to reduce the incidence of stress fractures in controlled trials. In contrast, strength and conditioning programs targeting the calf musculature, hip abductors, and core stabilizers may improve shock absorption, neuromuscular control, and load distribution, thereby reducing bone strain during impact activities [64].

### 7.5. Pharmacological Prevention

Pharmacological interventions have not demonstrated a preventive role in healthy athletic populations. Despite a theoretical rationale for reducing bone turnover, bisphosphonates have failed to prevent stress fractures in randomized controlled trials and may impair physiological microdamage repair by excessively suppressing bone remodeling [51]. Consequently, bisphosphonates should not be used for primary prevention of SFs in athletes.

### 7.6. Early Identification and Multidisciplinary Strategies

Effective prevention programs should incorporate regular athlete monitoring, early screening for RED-S, nutritional assessment, biomechanical evaluation, and systematic tracking of training load through logs or wearable technologies [9]. A multidisciplinary approach involving sports physicians, strength and conditioning coaches, sports dietitians, and psychologists is particularly important in high-risk sports, where cumulative loading and psychosocial stressors frequently coexist (Table 5).

## 8. Conclusions

Stress fractures represent a frequent and clinically relevant challenge in athletic populations, arising from the interaction between repetitive mechanical loading, bone biology, and modifiable intrinsic and extrinsic risk factors. Early recognition of bone stress injuries, accurate risk stratification based on MRI severity and anatomical site, and timely implementation of individualized management strategies are essential to prevent progression, reduce recurrence, and optimize return to sport.

Conservative management remains effective for most low-risk lesions, while high-risk stress fractures require closer monitoring and, in selected cases, early surgical intervention. Prevention strategies should prioritize training load optimization, biomechanical assessment, adequate energy availability, and early identification of RED-S, supported by a coordinated multidisciplinary approach. Integrating clinical evaluation with imaging-guided decision-making allows sports medicine clinicians to deliver safe, evidence-based, and sustainable care for athletes across performance levels.

## Figures and Tables

**Table 1:** Most Common Stress Fractures According to Sport-Specific Demands.

Sport/Activity	Most Frequently Involved Sites	Predominant Biomechanical Mechanism
Military training	Tibia, metatarsals	Sudden load increase, repetitive marching/running
Long-distance running	Tibia, metatarsals, femur	Repetitive impact loading
Basketball	Fifth metatarsal, tibia	Jump–landing cycles, cutting, acceleration
Football (soccer)	Fifth metatarsal, tibia	Cutting, sprinting, artificial turf exposure
Tennis	Navicular, pars interarticularis, humerus	Multidirectional loading, rotational stress
Dance (ballet/contemporary)	Second metatarsal, tibia	Forefoot overload, repetitive impact
Track & field (jumpers)	Anterior tibia, navicular	High tensile stress, plyometric loading
Gymnastics	Pars interarticularis	Repetitive lumbar hyperextension
Rowing	Ribs	Repetitive trunk loading, muscle fatigue
Golf	Ribs	Rotational stress, asymmetric loading
Adolescent athletes	Tibia, pars interarticularis	Skeletal immaturity, rapid growth

**Table 2:** Extrinsic and Intrinsic Risk Factors for Stress Fractures in Athletes.

Extrinsic Factors	Intrinsic Factors
Sudden increase in training volume, intensity, or frequency	Female sex
Inadequate load progression/poor training periodization	Low energy availability/RED-S
Insufficient recovery between training sessions	Menstrual dysfunction/hormonal disturbances
Training on hard, irregular, or sloped surfaces	Low bone mineral density
Inappropriate, worn-out, or poorly cushioned footwear	Delayed menarche
Abrupt change in training surface or footwear	Biomechanical and anatomical variants (pes cavus/planus, malalignment)
High-impact or weight-sensitive sports	Low muscle mass
Repetitive high-impact or plyometric loading	History of eating disorders
Previous stress fracture	Previous stress fracture

**Table 3:** MRI-Based Severity Grading of Bone Stress Injuries (adapted from the Kaeding–Miller classification).

Grade	MRI Findings	Typical Clinical Presentation
**Grade 1**	Periosteal or bone marrow edema on fluid-sensitive sequences (T2/STIR) without cortical involvement or fracture line	Minimal or no pain, usually activity-related
**Grade 2**	More extensive bone marrow edema without a visible fracture line	Localized pain during activity
**Grade 3**	Bone marrow edema with a visible, nondisplaced fracture line	Pain during activity and daily activities
**Grade 4**	Displaced fracture line or complete cortical disruption	Persistent pain, often at rest

**Table 4:** Anatomical Risk Classification of Stress Fractures.

Low-Risk Sites	High-Risk Sites
Fibula	Femoral neck (especially tension side)
Calcaneus	Anterior tibial cortex
Cuboid	Navicular bone
Cuneiforms	Talar body
Posteromedial tibia	Proximal fifth metatarsal (Jones fracture)
Second–fifth metatarsal shafts	Base of second metatarsal
Medial malleolus	Sesamoids
Rib	Pars interarticularis
Pelvis (pubic rami, sacrum—compression side)	Scaphoid

**Table 5:** Evidence-Based Prevention Strategies for Stress Fractures in Athletes.

Prevention Strategy	Clinical Rationale	Level of Evidence
Risk factor identification	Screening for intrinsic (biomechanics, RED-S, hormonal disturbances, low BMD) and extrinsic (training load, surface, footwear) contributors	Level II–III
Training load optimization	Gradual progression of volume and intensity; avoidance of rapid load spikes; structured recovery	Level II
Biomechanical assessment	Identification and correction of malalignment, movement inefficiencies, and kinetic-chain deficits	Level II–III
Footwear and insoles	Appropriate shoe selection and timely replacement; cushioned insoles are beneficial mainly in military settings	Level I–II
Nutritional optimization	Adequate energy availability; sufficient intake of calcium, vitamin D, protein, and micronutrients	Level II
RED-S screening	Early identification and management of low energy availability and endocrine suppression	Level III
Multidisciplinary monitoring	Integrated approach involving medical, nutritional, biomechanical, and psychological expertise	Level III–IV

## Data Availability

No new data were created or analyzed in this study. Data sharing is not applicable to this article.
